# ASAP: A new global early warning system to detect anomaly hot spots of agricultural production for food security analysis

**DOI:** 10.1016/j.agsy.2018.07.002

**Published:** 2019-01

**Authors:** Felix Rembold, Michele Meroni, Ferdinando Urbano, Gabor Csak, Hervé Kerdiles, Ana Perez-Hoyos, Guido Lemoine, Olivier Leo, Thierry Negre

**Affiliations:** European Commission, Joint Research Centre, Directorate D – Sustainable Resources, Via E. Fermi 2749, I-21027 Ispra, VA, Italy

## Abstract

Monitoring crop and rangeland conditions is highly relevant for early warning and response planning in food insecure areas of the world. Satellite remote sensing can obtain relevant and timely information in such areas where ground data are scattered, non-homogenous, or frequently unavailable. Rainfall estimates provide an outlook of the drivers of vegetation growth, whereas time series of satellite-based biophysical indicators at high temporal resolution provide key information about vegetation status in near real-time and over large areas. The new early warning decision support system ASAP (Anomaly hot Spots of Agricultural Production) builds on the experience of the MARS crop monitoring activities for food insecure areas, that have started in the early 2000's and aims at providing timely information about possible crop production anomalies. The information made available on the website (https://mars.jrc.ec.europa.eu/asap/) directly supports multi-agency early warning initiatives such as for example the GEOGLAM Crop Monitor for Early Warning and provides inputs to more detailed food security assessments that are the basis for the annual Global Report on Food Crises. ASAP is a two-step analysis framework, with a first fully automated step classifying the first sub-national level administrative units into four agricultural production deficit warning categories. Warnings are based on rainfall and vegetation index anomalies computed over crop and rangeland areas and are updated every 10 days. They take into account the timing during the crop season at which they occur, using remote sensing derived phenology per-pixel. The second step involves the monthly analysis at country level by JRC crop monitoring experts of all the information available, including the automatic warnings, crop production and food security-tailored media analysis, high-resolution imagery (e.g. Landsat 8, Sentinel 1 and 2) processed in Google Earth Engine and ancillary maps, graphs and statistics derived from a set of indicators. Countries with potentially critical conditions are marked as minor or major hotspots and a global overview is provided together with short national level narratives.

## Highlights

• Earth Observation based global monitoring of crop and rangeland conditions for food security analysis
• Timely early warning thanks to frequent updates (10 days for climate anomalies, 30 days for agricultural hotspot analysis)
• Public online webGIS with downloadable information
• Country level agricultural production hotspot identification and short narratives for non-remote sensing experts

## 1 Introduction

Agricultural production follows strong seasonal patterns related to the biological life cycle of crops and rangelands and it is at the same time dependent on climatic drivers and on physical characteristics of the landscape. This complexity, together with the challenges added by trends in human population growth, environmental degradation and climate change, continue to increase the needs for timely agricultural information. In this framework, agricultural monitoring is relevant to detect short-term deficits in crop production in response to weather variability but also to facilitate longer-term rural development, especially in areas of the world affected by a high risk of food insecurity. As a result, a number of monitoring systems exists at the global and regional scales (Fritz et al., 2018).

Information on crop and rangeland conditions ultimately facilitates risk reduction and leads to improved statistical analyses at a range of scales, enabling a timely and accurate national to sub-national agricultural statistical reporting that can be used to plan policies that prevent or mitigate food crisis. Recent examples of food production oscillations threatening food security include the 2015–2016 El Niño event with its strong negative impact on agriculture in both Eastern and Southern Africa (FAO, 2016) as well as the latest drought affecting the Horn of Africa in 2016–2017 (FAO, 2017).

Remote sensing can significantly contribute to agricultural monitoring as it allows gathering information about the biophysical state of vegetation over large areas with high revisit frequency (Atzberger et al., 2016). Crop development and growth can be monitored from space using spectral information in particular in the visible and near-infrared reflected domain (Rembold et al., 2016a), although other spectral regions are also relevant (e.g. the short-wave infrared for estimating vegetation water content, Colombo et al., 2011). Global coverage has been provided for many years by moderate spatial resolution (250 to 1000 m) satellite instruments with daily revisit at global level (e.g. Moderate-resolution Imaging Spectroradiometer, MODIS; Satellite Pour l'Observation de la Terre, SPOT-VEGATATION). High spatial resolution imagery (10–20 m) with high temporal frequency (5 days) is also becoming available thanks to the Copernicus program (Sentinel 1 and 2) but does not have a long time series yet, which limits their use in many crop monitoring applications that typically compare the current situation to the past.

A pragmatic and widespread approach to extract the relevant information from the various spectral bands of satellite sensors relies on the computation of vegetation indices (VIs) that are mathematical combinations of spectral bands used to quantify vegetation amounts and vigour. Among the different VIs, the Normalized Difference Vegetation Index (NDVI; Rouse et al., 1974) based on the red and near-infrared reflectances is often employed for studying vegetation health and crop production.

To detect unfavourable growth conditions before crop development is affected, the main meteorological drivers of vegetation growth (i.e. precipitation, temperature, incident radiation) can also be monitored. In particular, in food insecure arid and semi-arid areas of the world, precipitation often represents the main limiting factor to vegetation growth. Among the various rainfall-based indexes that have been proposed for drought monitoring, the Standard Precipitation Index (SPI, World Meteorological Organization, 2012) is commonly employed to track precipitation anomalies. SPI can be compute from rainfall stations data. However, the sparse and irregular network of rainfall station makes regional and global analysis difficult. Gridded global rainfall estimates are generated by atmospheric circulation models (e.g. rainfall product from the European Centre for Medium-Range Weather Forecasts, ECMWF, 2015) or derived from meteorological satellite observations and rainfall station data (e.g. CHIRPS, Funk et al., 2015).

At present, multi-decadal archives of continuous observations are available for both satellite vegetation indexes and rainfall estimates, making time series analysis a common tool for drought monitoring (Rembold et al., 2016a). Typically, the first step in the crop and rangeland early warning context is the computation of rainfall or VI anomalies. For instance, by computing the anomaly of the current NDVI observation, the actual current vegetation status is compared to previous seasons or to what can be assumed to be the average or normal situation. Detected anomalies are then used to draw conclusions on vegetation status and yield limitations. As anomalies at different locations may refer to different stages of development, the analysis has to include information on the actual development stage of the vegetation. Information about temporal vegetation phenology can be retrieved from satellite VI time series and used for a correct interpretation of observed anomalies (e.g. Meroni et al., 2014a; Meroni et al., 2014b).

Starting in 2001, the Monitoring Agricultural Resources unit (MARS) of the Joint Research Centre (JRC) has applied techniques developed for crop monitoring in Europe to areas with high risk of food insecurity (Rojas et al., 2005). The first operational product in a series of national and regional crop monitoring bulletins for the food security analysis community was focussing on Somalia. It consisted of a report issued every 10 days on crop conditions based mainly on remote sensing information. The regular analysis continued until 2006, when the methodology was fully integrated into the capacity of the Food Security and Nutrition Analysis Unit - Somalia project (Massart et al., 2010). Similar regular reports were also produced for many years for Ethiopia, Eritrea (still active) and, at the regional level, for East and West Africa. After 2010 and on request of partner Directorate Generals of the European Commission (EC) and of European Union (EU) Delegations, these bulletins were produced on an ad hoc basis, during or after food security crises. This was the case for example for Niger, Kenya, North Korea, Angola, Zimbabwe, Namibia and several others. At the same time, there was also a transition towards including more information about other dimensions of food security, going beyond crop production monitoring. In recent years in fact there has been a trend for harmonized food security assessments (for example thanks to the Integrated Food Security Phase Classification initiative, IPC Global Partners (2012), and the Cadre Harmonisé, AGRHYMET (2014)) and for multi-agency reports such as the Global Report on Food Crises (Food Security Information Network, 2017). These approaches combine and harmonize information from different sources other than agronomic data (e.g. economic and nutrition surveys, prices information, conflicts information) and provide a higher level of synthesis to end users and policy makers. In addition, global agricultural production monitoring initiatives have been launched such as the Group on Earth Observations Global Agricultural Monitoring (GEOGLAM) initiative launched by the G20 international forum in 2011. These collaborative actions provide consistent and timely information at the global level based on contributions by their members.

To provide early warning information at the global level on a near real time basis, MARS recently developed and launched a new on-line early warning system called ASAP (Anomaly hot Spots of Agricultural Production). ASAP capitalizes on global datasets of weather and vegetation data from models and remote sensing observations and on well-established time series analysis methods. The system makes available timely overviews of production anomalies at the global level as input to more detailed agricultural monitoring or food security assessments. In this way, it complements the quantitative crop monitoring and yield forecasting analysis provided by MARS for Europe and its neighbouring countries and summarized in the MARS bulletins (Baruth et al., 2018). This integration with the MARS Crop and Yield Forecasting System (MCYFS, Genovese et al., 2004) is both geographic and thematic, since ASAP provides global early warning information with a focus on food insecure countries as opposed to the detailed agricultural monitoring and yield forecasting for Europe and neighbouring countries included in the MARS bulletins. The user communities are also clearly distinguished with the MARS bulletins serving primarily the Directorate General for Agriculture and Rural Development and the EU Member States, and ASAP addressing the Directorate General for International Cooperation and Development in charge of programming the food security related assistance for the EC, the EU delegations in food insecure countries, and the international multi-agency initiative GEOGLAM - Crop Monitoring for Early Warning (CM4EW, https://cropmonitor.org/), as well as regional and national food security analysts across the globe.

In particular, ASAP provides information at two levels: 1) ten-day automatic warnings at sub-national level, and 2) monthly country assessments with the identification of agricultural production hotspots and summary narratives by JRC experts. The automatic warnings are generated globally for crop and rangeland areas. The warnings classification system is described in details in 2, 3, while in Section 5 some examples of its outputs are provided. The warnings, together with input indicator maps and a set of additional graphs and statistics at sub-national level, are made available on-line in the ASAP Warning Explorer (https://mars.jrc.ec.europa.eu/asap/hsds/) for users with expertise in geospatial data.

The expert-based assessment at national level targets 80 selected countries included the list of food insecure countries monitored by the GEOGLAM-CM4EW and additional countries where food security and rural development are target sectors of the European Development Fund. It involves the analysis of the automatic warnings together in conjunction with additional information, in particular high spatial resolution remote sensing data, news from the press and a large set of maps, graphs and statistics generated by the ASAP system (see Section 4). Agricultural analysts identify the countries with production deficits possibly leading to increased risk of food insecurity and mark them as minor or major hotspots. The map of the current hotspot countries, a global overview and country reports that includes short early warning messages by MARS analysts are published on the ASAP Hot Spot web page (https://mars.jrc.ec.europa.eu/asap/).

## 2 Data and geographical settings

### 2.1 NDVI and rainfall estimates

The analysis is performed on NDVI data from the MetOp mission (operated by the European Organization for the Exploitation of Meteorological Satellites, EUMETSAT) at 1 km spatial resolution available form year 2007. The NDVI is computed from top-of-canopy red and near-infrared reflectances obtained using the SMAC atmospheric correction algorithm (Rahman and Dedieu, 1994) applied to daily images of the AVHRR/3 instrument on board of the MetOp-A platform. Ten-day maximum NDVI composites (Holben, 1986) are then produced and temporally smoothed with the Swets algorithm (Swets et al., 1999).

Rainfall estimates (RFE) are gathered from the European Centre for Medium-Range Weather Forecasts (ECMWF) forecasting system. Compared to other data sources, ECMWF models additionally provide near real-time estimation of the air temperature, that is used for the estimation of potential evapotranspiration component of the water requirement satisfaction index (GWSI) an indicator of crop and rangeland water stress that will be incorporated in ASAP in the near future (see Section 6). The time series of ERA-Interim reanalysis model is used for the period spanning from 1989 up to 2015. Era-Interim variables are produced at 6-hourly time-step at a spatial resolution of approximately 80 km. Data from 2016 up to the time of analysis are from the high-resolution forecast model (HRES), originally produced (at 00 and 12 UTC) with a 3-hourly time-step and approximately 9 km spatial resolution (ECMWF, 2015) and then gridded to a 0.25° resolution. While HRES forecasts are produced for the next 10 days, only those of the first day of this 10-day forecast depth, considered more reliable estimates of actual precipitation, are retained here and used to compute daily precipitation values. After computation of daily values, ERA-Interim is then scaled to the reference grid of HRES and temporally aggregated to 10-day cumulative precipitation values. Different models are used because the ERA-Interim reanalysis model is not publicly available in near real-time.

To match two different resolutions used in the warning classification system (1 km NDVI and 0.25° ECMWF RFE), the coarser resolution data is resampled to the 1 km grid using nearest neighbour resampling.

### 2.2 Crop and rangeland masks

Cropland and rangeland areas in Africa are identified using masks generated from the land cover/land use dataset of Vancutsem et al. (2013). For the rest of the world we used the GlobCover 2005–06 (Bicheron et al., 2008) with the exception of following countries/regions where we used more specific land use maps: Afghanistan (Land Cover of Afghanistan 1993; FAO, 1993), Argentina (Cobertura del suelo de la Republica Argentina 2006–07; Volante, 2009), Australia (National scale land use 2001–02; Bureau of Rural Sciences, 2006), Europe (Corine land cover map 2000; Bosard et al., 2000), Mexico (MODIS land cover classification; Giri and Jenkins, 2005), U.S.A. (National Land Cover Database of United States; Homer et al., 2004).

The masks, derived from cropland and rangeland maps with resolution of 250 m (Vancutsem et al., 2013), are expressed at the lower spatial resolution of NDVI data (1 km) as area fraction images (AF, i.e. the percentage of the pixel occupied by crop and rangeland, ranging from 0 to 100%).

### 2.3 Geographical domain

The warning classification scheme is applied globally at the first sub-national administrative level, i.e. GAUL level 1, Global Administrative Units Layers of the Food and Agriculture Organization (FAO) of the United Nations (FAO, 2014). This level was identified as a compromise with respect to the trade-off between the need of analysing units with homogeneous agro-ecological characteristics (ideally small units) vs. the need of summarizing the results for a global outlook (ideally large units). In addition, working with administrative units has the advantage that they are well recognized and analysts can easily compare with other data normally available at the administrative level (crop types, calendars, area and yield statistics, etc.).

The GAUL1 units have been adapted to the specific needs of the early warning system with minor modifications (e.g. suppression/aggregation of negligibly small units, split of large and agro-climatically heterogeneous units, and exclusion of units with irrelevant crop or rangeland area). The modified units will hereafter referred to as ASAP units.

### 2.4 Identification of water-limited units

Water, temperature and radiation are the main limiting factors to vegetation growth at the global level (Nemani et al., 2003). All limiting factors are indirectly covered by the NDVI series used in ASAP, which allows to spot sub-optimal vegetation growth, independently from the drivers. Nevertheless, with ASAP we also monitor precipitation deficit with the aim of anticipating biomass development problems. However, as the interpretation of precipitation anomalies in non water-limited areas is not straightforward and may be misleading, the use of precipitation-related indicators in the warning classification system is restricted to water-limited regions. To define water-limited regions, we used a simplified annual climatic water balance, represented by the difference between the mean cumulative annual values of precipitation and potential evapotranspiration, similarly to the aridity index of the United Nations Environment Programme (Middleton and Thomas, 1997). Both precipitation and potential evapotranspiration are from ECMWF ERA-Interim over the period 1989–2015. A negative water balance indicates regions where water is a limiting factor, i.e. the evaporative demand exceeds precipitation.

### 2.5 Data processing

Imagery data processing is mainly based on the free software SPIRITS (Eerens et al., 2014; Rembold et al., 2015) in combination with the open source tools GDAL 11.1 (Warmerdam, 2008) and POSTGIS 2.3 (Obe and Hsu, 2011). Data are managed using PostgreSQL 9.5(https://www.postgresql.org/). Graphs are generated by R 3.12 (R Core Team, 2016). Web tools are developed using mainly Geoserver (http://geoserver.org/), Openlayers (https://openlayers.org/) and highcharts (www.highcharts.com). The whole processing chain is automated using JAVA (https://www.java.com/) and Python scripts (Python Core Team., 2016).

## 3 Methods

The warning classification system for crops and rangelands is updated every 10 days when a new NDVI temporal composite and ten-day rainfall cumulates are made available. For simplicity and conciseness, hereafter we will only refer to croplands in the explanation of the methodology that is applied without modification to rangelands as well. The basic indicators used in the system are NDVI and RFE anomalies. Although anomalies can be computed at any location and any time of the year, they are relevant in agronomic sense only where and when crops are growing. Whereas crops are spatially identified by the mask described in Section 2.2, their average growing season is defined by satellite-derived phenology.

### 3.1 Satellite-derived phenology

To define the mean growing season period we use the satellite-derived phenology computed on the long-term average of 10-day SPOT-VEGETATION NDVI time series (average temporal evolution computed over the period 1999–2013). The software uses an approach based on thresholds on the green-up and decay phases as described in White et al. (1997).

The following key parameters are retrieved for each pixel: number of growing seasons per year (i.e. one or two); start of season (SOS, i.e. the time when NDVI rises above 25% of the ascending amplitude of the seasonal profile); time of maximum NDVI (TOM); start of senescence period (SEN, when NDVI drops below 75% of the descending amplitude); and end of the season (EOS, when NDVI drops below 35%).

With this information it is then possible to determine, at any time of analysis, if a pixel is “active” (i.e. in the period of average growing season) and to compute the progress of the season and the phenological stage. The progress of the season is the percentage of the length of the growing season (i.e., EOS minus SOS) that has passed at the time of analysis. A progress of 50% thus indicates that the pixel is half-way through the season. The period between SOS and TOM is referred to as phenological stage “expansion”, the one between TOM and SEN as “maturation”, and the one between SEN and EOS as “senescence”.

### 3.2 Pixel level analysis

#### 3.2.1 Computation of anomalies

Remote sensing and meteorological data are first analysed at the pixel level by computing anomalies. We use the cumulative value of NDVI over the growing season, a proxy of biomass production (Prince, 1991; Tucker et al., 1985) and crop yield (e.g. Funk and Budde, 2009; Meroni et al., 2013). At time of analysis t = T (being t the ordinal 10-day period during the year, t = [1, 36]), the cumulative value of NDVI (NDVIc) and the derived anomalies are defined by:

(1) $$\mathrm{NDVIc}\left(T\right)=\sum_{t=\mathrm{SOS}}^{T}\mathrm{NDVI}\left(t\right)$$

(2) $$\mathrm{zNDVIc}\left(T\right)=\frac{\mathrm{NDVIc}\left(T\right)-\mu_{\mathrm{NDVIc}}\left(T\right)}{\sigma_{\mathrm{NDVIc}}\left(T\right)}$$

(3) $$\mathrm{mNDVId}\left(T\right)=\frac{\mathrm{NDVIc}\left(T\right)-\mu_{\mathrm{NDVIc}}\left(T\right)}{n}$$

where SOS is the start of season, μ_NDVIc_(T) and σ_NDVIc_(T) are the multi-annual mean and its corresponding standard deviation of NDVIc at time T, and n is the number of 10-day periods from SOS to T. Mean and standard deviation values are computed over the historical multi-annual archive of NDVI observations and updated at the beginning of each calendar year. zNDVIc is a standardized anomaly (z-score) expressing the current NDVIc deviation from the mean value in standard deviation units. mNDVId is the mean difference of NDVI (i.e. current value minus historical average).

RFE data are used to compute the Standardized Precipitation Index (SPI, World Meteorologic Organization, 2012), an index widely used to characterise meteorological drought at a range of timescales. The SPI is a probability index that expresses the observed cumulative rainfall for a given time scale (i.e. the period during which precipitation is accumulated) as the standardized departure from the rainfall probability distribution function. The frequency distribution of historic rainfall data for a given pixel and time scale is fitted to a gamma distribution and then transformed into a standard normal distribution. We computed the SPI using data from 1989 to current date and two accumulation periods: one and three months. SPI1 and SPI3 (i.e. using 1 and 3 months accumulation period) are considered to account for a short and prolonged meteorological water shortage, respectively.

#### 3.2.2 Flagging critical anomalies

To determine the fraction of the crop area in each ASAP unit that is affected by a severe anomaly, we proceed as follows. Pixel level standardized anomalies (SPI1, SPI3, and zNDVIc) are flagged as “critical” when smaller than −1 (i.e. negative anomaly larger than one standard deviation). Under the assumption of normal distribution, 16% of the observed values would be smaller than −1.

In this way, each pixel in the ASAP unit is flagged as critical (or not) for SPI1, SPI3 and zNDVIc. To avoid flagging as critical those vegetated pixels with small NDVI inter-annual variability we also consider the mean of the difference between NDVI and its long term average over the growing season (mNDVId). Thus, pixels having a zNDVIc value smaller than −1 are flagged as critical only if also mNDVId < −0.05. Symmetrically, we also consider large positive anomalies (zNDVIc >1 and mNDVId >0.05) to flag the pixel as “exceptionally favourable conditions”. The combination of zNDVIc and mNDVId is called zmNDVI.

### 3.3 ASAP unit level analysis

#### 3.3.1 Spatial domain

Warnings are issued at the ASAP unit level. Pixel-level information is summarized at this level, computing the fraction of the unit area that is affected by different critical anomalies (see Section 3.3.3). We analyse cropland and rangeland areas separately. Anomalies occurring outside such targets are not considered. All area-based calculations are made taking into account the area fraction of cropland and rangeland in each pixel using AF images. For instance, the extent of the crop area is the sum of the area of the crop pixels weighted by their AF.

#### 3.3.2 Temporal domain

Anomalies are computed only for times when crops are growing. Although we use static crop AF images as a base layer, we “switch on and off” the property of being an active crop at the pixel-level, according to the pixel phenology. In this way, we obtain 36 crop masks, one per each 10-day interval of the year, indicating per pixel the presence of crop in its growing season period. In order to focus the analysis over the main growing season period of the ASAP unit, the classification is performed only when the time of analysis is within the multi-annual average period of the growing season for at least 15% of the total crop area.

Mono- and bi-modal seasons (i.e. one and two growing cycles per solar year, respectively) may be present within an ASAP unit. Although a dominance of one of the two modalities can be expected, both of them can be present at the same time. As a reference for the entire unit, we compute the median progress of the season (in %) and the modal phenological stage (expansion, maturation and senescence). This “merging” of the two seasons was conceived in order to avoid treating mono- and bi-modal separately, with the consequence of doubling the targets (i.e. crop/rangeland, mono−/bi-modal) and making results more difficult to be interpreted by the user.

#### 3.3.3 Computation of the fraction of critical area

The warning level is based on the fraction of the area (of crop pixels having an ongoing growing season) being subjected to the different critical anomalies (SPI1, SPI3, and zmNDVIc). This geographic aggregation scheme (Rojas et al., 2011) aims at detecting unfavourable growing conditions that affect a significant fraction of the crop area and may thus lead to a food security problem. We thus trigger a warning only if two conditions on the anomalies are met: i) the negative anomaly of at least one indicator is severe (Section 3.2.1) and ii) it affects a significant extent of the active area in the ASAP unit. It is noted that by taking the overall spatial mean of the anomaly we would instead mix the two components. For instance, a negative anomaly affecting half of the crop area when the remaining area shows a positive anomaly would result in a spatial average showing normal conditions.

We compute the critical area fraction (CAF) as the ratio between the crop area flagged as critical and the total crop area with an active growing season at time of analysis:

(4) $${\mathrm{CAF}}_{x}=\frac{\mathrm{critical}\_{\mathrm{area}}_{x}}{\mathrm{active}\_\mathrm{area}}$$

The subscript x refers to the indicator considered (i.e. SPI1, SPI3, zNDVIc). In addition to the CAF of each single indicator, we also consider the CAF_u_, computed with a critical area composed by the spatial union of the areas flagged by each indicator, and the CAF_e_, determined by zNDVIc area flagged as exceptionally favourable.

#### 3.3.4 Warning classification

When one or more CAFs exceed a threshold of 25% (i.e. one quarter of the active crop area) a warning is triggered for the ASAP unit. To avoid triggering a warning when CAF is above the threshold but represents only a small area that has very limited potential impact on food security, we retain the warning only if the active area is larger than 100 km^2^.

The specific warning level is determined by the type and combination of CAFs exceeding the threshold. We put emphasis on the reliability of the various indicators and their agreement. We acknowledge that for the water-limited areas considered in ASAP, rainfall is the main driver of crop and rangeland growth. An observed deficit may thus affect future crop development. The longer the deficit, the higher the likelihood of a detrimental effect. NDVI anomalies show instead that biomass development is indeed affected by drought (or other perils like pests or flooding). Thus, we rank the NDVI anomaly events with higher warning level (level 2) compared to those due to RFE alone (level 1, Table 1). Finally, the occurrence of anomaly events generated by different indicators is ranked with level 3, the highest level reachable before the senescence phase.

**Table 1:**
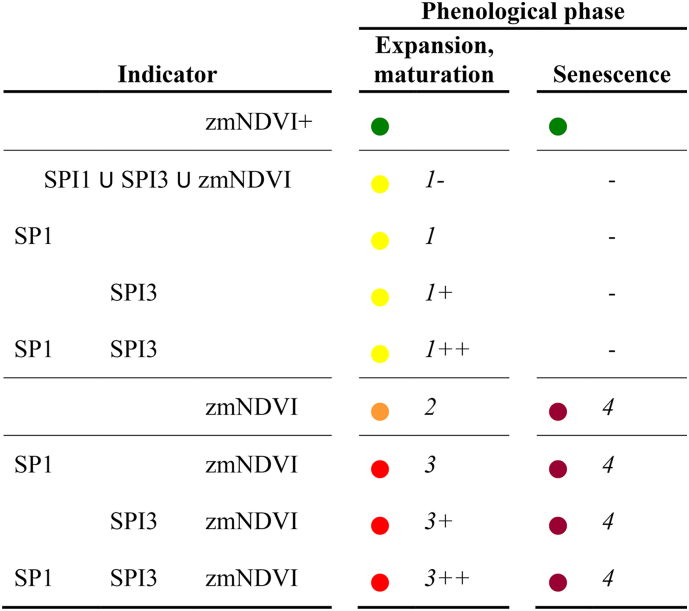
ASAP warning levels as a function of the individual indicators with CAF > 25% and phenological phase at which the warning occurs. The symbol U represents the spatial union operator used to compute CAF_u_. zmNDVI stands for zNDVIc AND mNDVId, both being flagged as critical. zmNDVI+ indicates that the CAF_e_ is exceeded (i.e. exceptionally favourable conditions). The legend used for mapping is depicted as coloured points.

The warning “1-” is the first level and it is triggered when the spatial union of the critical areas of all the three indicators exceeds the threshold of 25%. That is, none of the CAF_x_ exceeds the area threshold, but the total area affected by at least one of critical indicators (CAF_u_) does.

Levels from 1 to 1++ are triggered by rainfall-based indicators. The lowest level in this group is triggered by a deficit in the last month (i.e. SPI1) while the intermediate level (1+) is triggered by a more prolonged deficit (i.e. during the last three months, SPI3). The highest level of the group (1++) is assigned to the co-occurrence of the two conditions: a relatively long lasting deficit (SPI3) that persists in the last month (SPI1). An increased warning level (2) is assigned to the NDVI indicator as it shows that the growth of the vegetation has been affected, regardless of the causes. The level 3 (ranging from 3 to 3++) is assigned to the co-occurrence of NDVI- and rainfall-based indicators with a similar logic that was used for the level 1 group.

The phenological stage has an effect on the warning level: during senescence, rainfall based indicators do not trigger a warning and only NDVI is used because rainfall shortage does not necessarily affect crop production during this stage. In addition, a different warning level is assigned to the NDVI anomaly during senescence to highlight that at poor seasonal performance evaluated towards the end of the season can be considered a confirmation of season failure rather than a warning.

The occurrence of a positive anomaly in the NDVI-based indicators is also represented, and labelled as “exceptionally favourable conditions”. It is noted that the same ASAP unit may present simultaneously an “exceptionally favourable condition” and a warning, for example in the case of spatially heterogeneous crop conditions within a large unit. The observed frequency of such co-occurrence is indeed negligible (<0.004% of total number of warnings).

## 4 Other information used for agricultural production hot spot identification

The final identification of hot spot countries by MARS analysts is based on the automatic ASAP warnings described in the previous paragraphs and on a set of additional information that includes high spatial resolution data, maps, statistics, graphs and relevant multilingual media information extracted using existing media monitoring techniques.

### 4.1 High resolution data

One of the main novelties of ASAP is the integration of high spatial and temporal resolution data.

Available Sentinel 2 and Landsat 8 data for any ASAP unit are retrieved from the Google Earth Engine catalogue (Gorelick et al., 2017). A viewer is implemented as a Google App Engine web application, which is directly linked to Google Earth Engine. For the ASAP unit of interest and a time-period, the viewer shows: i) a near-infrared false colour composition for the selected period and the same period of the previous year; and ii) the NDVI difference between the two periods.

The user can select a compositing period length, its ending date and maximum image cloud cover fraction. Default values are set to a monthly composite ending the day of the last assessment, with 10% maximum cloud cover. The images acquired over the selected period are composited and mosaicked over the ASAP unit of interest as single images of near-infrared false colour composition and NDVI. The minimum operator is selected to temporally composite the individual bands of the false colour image. The maximum operator is used to temporally composite the NDVI images. The false colour and NDVI composites are generated for both instruments (Sentinel 2 and Landsat 8), the selected period and the same period of the previous year. The NDVI is displayed as a difference image (current year minus the previous year).

An example is provided in Fig. 5. The visualization of high resolution imagery adds relevant information for analysis such as vegetation cover and vigour at field level for the month prior to analysis. This viewer is, for the time being, the only platform providing a graphic user interface to quickly access Landsat and Sentinel monthly RGB composites and NDVI differences with the previous year, mosaicked for the administrative region of interest.

### 4.2 Statistics, maps and graphs

A large set of summary statistics, relevant maps and graphs are produced at the country level every ten days and made available to analysts through a web-based interactive dashboard that supports the analyst's work of identifying whether a country should be classified as a hotspot. The full set of such information is not available to external users.

ASAP statistics at country level include: area subject to critical anomalies for SPI1, SPI3 and zmNDVI expressed as area (km^2^), percentage of active area and percentage of total area; crop and rangeland total area (km^2^) and active area (km^2^ and fraction of total area in percentage) at the time of the analysis; and history of previous monthly country hotspot classifications.

ASAP graphs include: the recent history of the warnings for all ASAP units in the country in matrix form (example provided in Table 2); active area fraction by anomaly range in crop and rangeland areas for NDVId, zNDVIc, SPI1 and SPI3 at national level; active area (km^2^) and fraction of active area (%) anomaly range in crop and rangeland areas for NDVId, zNDVIc, SPI1 and SPI3 in the ten most relevant ASAP units (example provided in Fig. 2a).

**Table 2:**
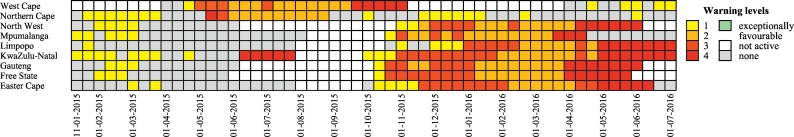
Table view of ASAP units for South Africa showing the 10-day warnings of the 18 months period ranging from 11 Jan. 2015 to 10 Jul. 2016.

**Fig. 1:**
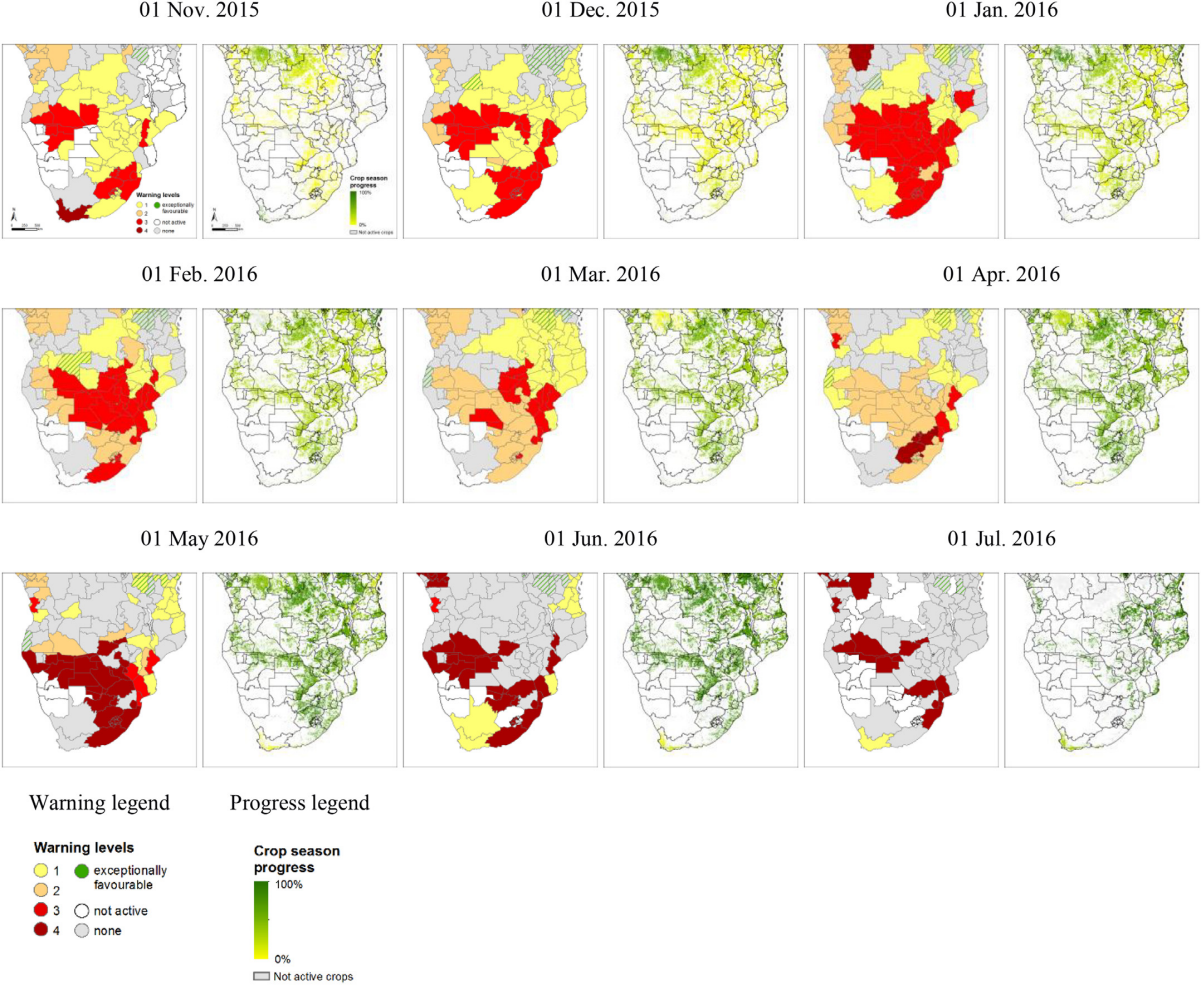
Temporal evolution of triggered warnings and progress of the season for the cropland areas in Southern Africa from November 2015 to July 2016. For each date the left and right panels represent the ASAP unit level warnings, and the pixel-level progress of the season, respectively. The reference date represents the first day of the 10-day period. Warning and progress legend are reported in the last row.

ASAP maps at country level include: a country overview of automatic warnings triggered at ASAP sub-national level for the reference dekad; crop and rangeland maps; and satellite-based phenology information. The latter includes: number of growing season per year; time of the start and end of the first and of the second season; progress of the season (an example is shown in the time series of Fig. 1); length of the first and of the second season. In addition, ASAP generates 10-day composite maps of the following indicators for the last two months: NDVI; zNDVIc; mNDVId; NDVId; SPI1; SPI3; zNDVI; rainfall; rainfall cumulated over the last month; and finally the difference anomaly (i.e. current value minus historical average) of the previous two rainfall variables.

A subset of the country-level information used by the analysts is made available to the public on the ASAP web site in the country report page when a new hotspot assessment is performed (i.e. every month). Additional statistics, graphs and indicators at ASAP sub-national level are available through the ASAP Warning Explorer (examples provided in Fig. 4).

### 4.3 Media monitor

Analysts can retrieve additional information by the multi-lingual media review provided by tailored queries (related to themes such as agriculture, drought, crisis, etc.) of the Europe Media Monitor (http://emm.newsbrief.eu/NewsBrief/alertedition/en/FoodSecurityFoodAid.html). This news analysis system gathers an average of 175,000 online news articles per day from about 4000 online sources in up to 75 languages (Steinberger, 2013). The specific strength of this system is the multi-language capability, allowing to pick up events of interest for countries that do not necessarily feature in international media.

## 5 Examples of recent ASAP warnings and agricultural production hot spot identification

### 5.1 Southern Africa 2015–2016

Southern Africa was affected by an intense drought related to a strong El Niño event during the 2015–2016 agricultural season. Fig. 1 shows the time series of the ASAP warnings for crops over the Southern Africa during the agricultural season of 2015–2016 depicting the evolution of the El Niño impact from its early appearance. Although the warning classification is performed every 10-days we report here the results at the monthly time-step for simplicity.

The temporal evolution of the impact depicted by ASAP warnings is in agreement with the information of various ground reports published later in the season (WFP, 2016; GEOGLAM CM4EW, 2016) and summarized hereafter.

Drought conditions in Southern Africa started in the third quarter of 2015 and rains, which normally mark the onset of the season in October, were delayed in many regions by one month. After the beginning of the rainy season, precipitation remained significantly below average for large parts of the region. This is visible in the ASAP warnings of 01 Nov. and 01 Dec., largely triggered by rainfall deficits (warnings level 1) and by the combination of NDVI and rainfall anomalies (warnings level 3). Mainly in the Eastern part of South Africa, Botswana, Namibia, Southern Angola, Zimbabwe, parts of Mozambique, Malawi and Zambia, the late onset of the season was followed by a dry and hot period until December. Between October and early February many of these areas had only received between 50 and 70% of their usual total cumulated seasonal rainfall (Rembold et al., 2016b). According to ASAP warnings, the maximum spatial extent of the area affected by biomass anomalies (triggered by zmNDVI, warning level 2 to 3) was achieved already in January. The Eastern part of Southern Africa received good quantities of rainfall during March, but this was mostly too late for crop conditions to recover. According to ASAP warnings, season failures (level 4 warning due to NDVI anomaly during the senescence phase) affected large part of this area. In South Africa the seasonal rainfall pattern, with initial strong deficits and late recovery, led to a total production that was about 40% below the 5 years average (WFP VAM, 2016).

The evolution of the event can be effectively monitored with the table view of the recent history of the warnings in the country (Table 2). The colour of each cell represents the warning level. The table columns (i.e. the Y-axis) represent the 10-day periods extending back one year and a half from the current date. The table rows (X-axis) are the ASAP units of the country.

Besides showing the impact of El Niño, Table 2 provides an overview of the seasonality of the different ASAP units. Taking the Free State unit as an example, it is possible to observe that the 2015 crop season started in October and was immediately affected by negative rainfall anomalies (warning level 1), followed by rainfall and NDVI anomalies (warning level 3) until the beginning of January. From January onwards the negative NDVI anomaly persisted although without further severe rainfall deficits (warning level 2). Due to the prolonged period of warnings, from early April, when the unit entered senescence, it was classified as failed season (warning level 4).

In the Eastern region of Mpumalanga a recovery of crop conditions due to late season rainfall is visible in Table 2, where the warnings stop at the end of April 2016. This is confirmed also by crop production statistics released by the Ministry of Agriculture in September 2016, where Mpumalanga's final maize production is less affected (−11,8% compared with the 2011–2013 average) as compared to the one of other main maize producing regions (−53,8% in Free State and − 25,1% in Gauteng) (Min. of Agriculture, Forestry and Fisheries, South Africa, 2016).

Finally it is interesting to observe how the warning progressed over the 2015/2016 season. In most cases, the sequencing of warning levels went from 1 via 3 and 2 to 4, meaning that the first warnings were triggered by rainfall deficits, then confirmed also by NDVI. With further progress of the relatively long season and with some late improvement of rainfall, the warnings are mainly due to below-normal vegetation productivity as shown by the negative NDVI anomalies.

**Fig. 2:**
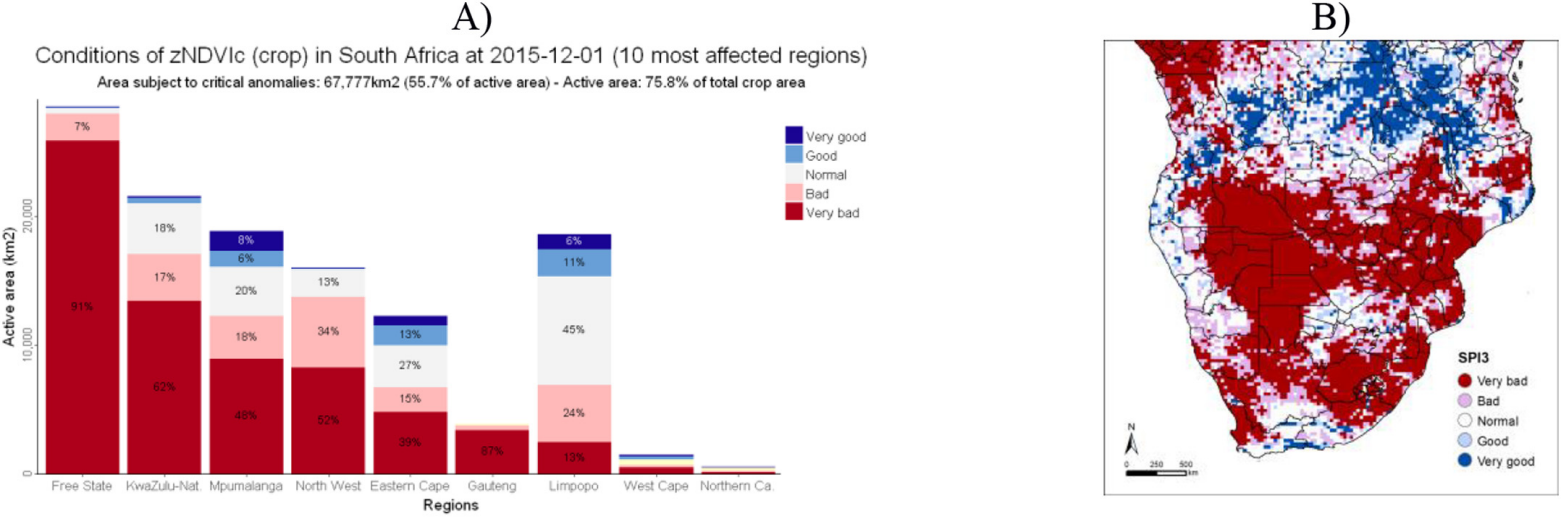
South Africa, 01 Dec. 2015. A): Active area and share of area affected by five classes of cNDVIz anomaly. B): SPI3 map. The numerical values for both legends (zNDVIc and SPI3) are: > 1 (Very good), 0.5:1 (Good), −0.5:0.5 (Normal), −1:-0.5 (Bad), and < −1 (Very bad).

As further examples of additional information generated by ASAP and that confirm the analysis above, Fig. 2 shows for South Africa the histogram of active area (and fraction) affected by different anomaly ranges of zNDVIc and the map of SPI1 at an early stage of the period affected by El Niño (1 Dec. 2015). At the time of the 2015 El Niño impact, only a prototype of the ASAP Early Warning Explorer was running and there was no ASAP analyst assessment. However, information from the Warning Explorer substantially contributed to the JRC analysis included in the report by Rembold et al., 2016b.

### 5.2 Coastal Kenya and Somalia 2016

**Fig. 3:**
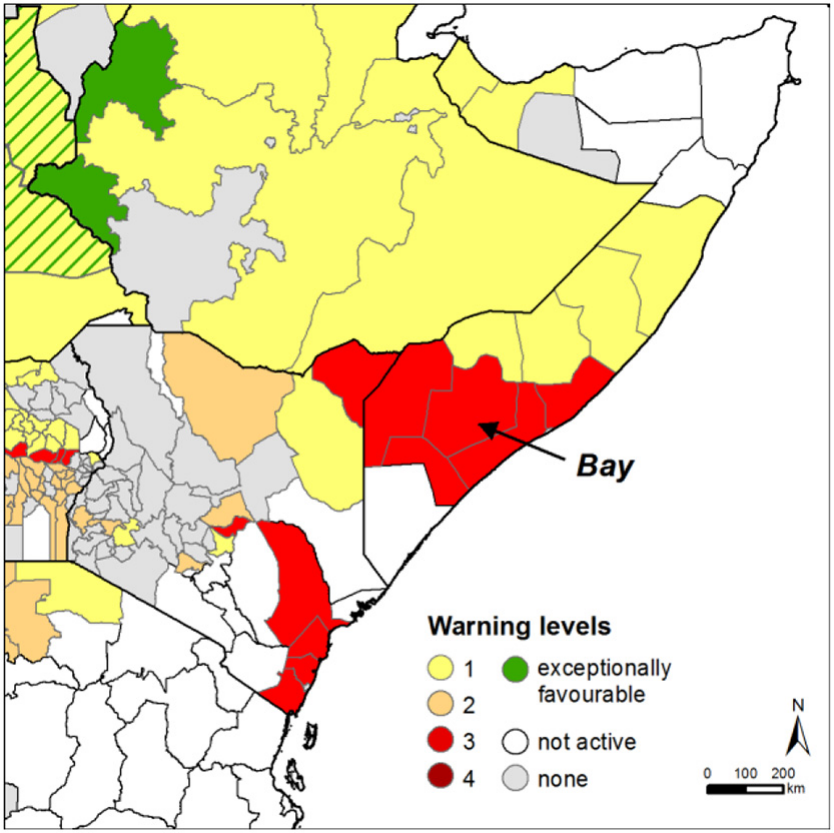
Warning map as of 20-Oct-2016 for Somalia, Kenya and Ethiopia.

Dry weather conditions during the short rainy season (approximately October–December) of 2016 have affected crop and rangeland productivity in large parts of East Africa. In Somalia, where the season extends roughly from October to January, rainfall-based warnings were issued for all the main agricultural areas of Southern Somalia since the beginning of October, whereas NDVI was affected the following 10-day period (Fig. 3).

In the coastal areas of Kenya this season corresponds to the main production season and similarly to Southern Somalia, the warnings started in the second 10-day period of October 2016 and remained in level 3 or moved to 4 until the end of the season in January/February 2017, in agreement with the analysis of WFP VAM (2017).

**Fig. 4:**
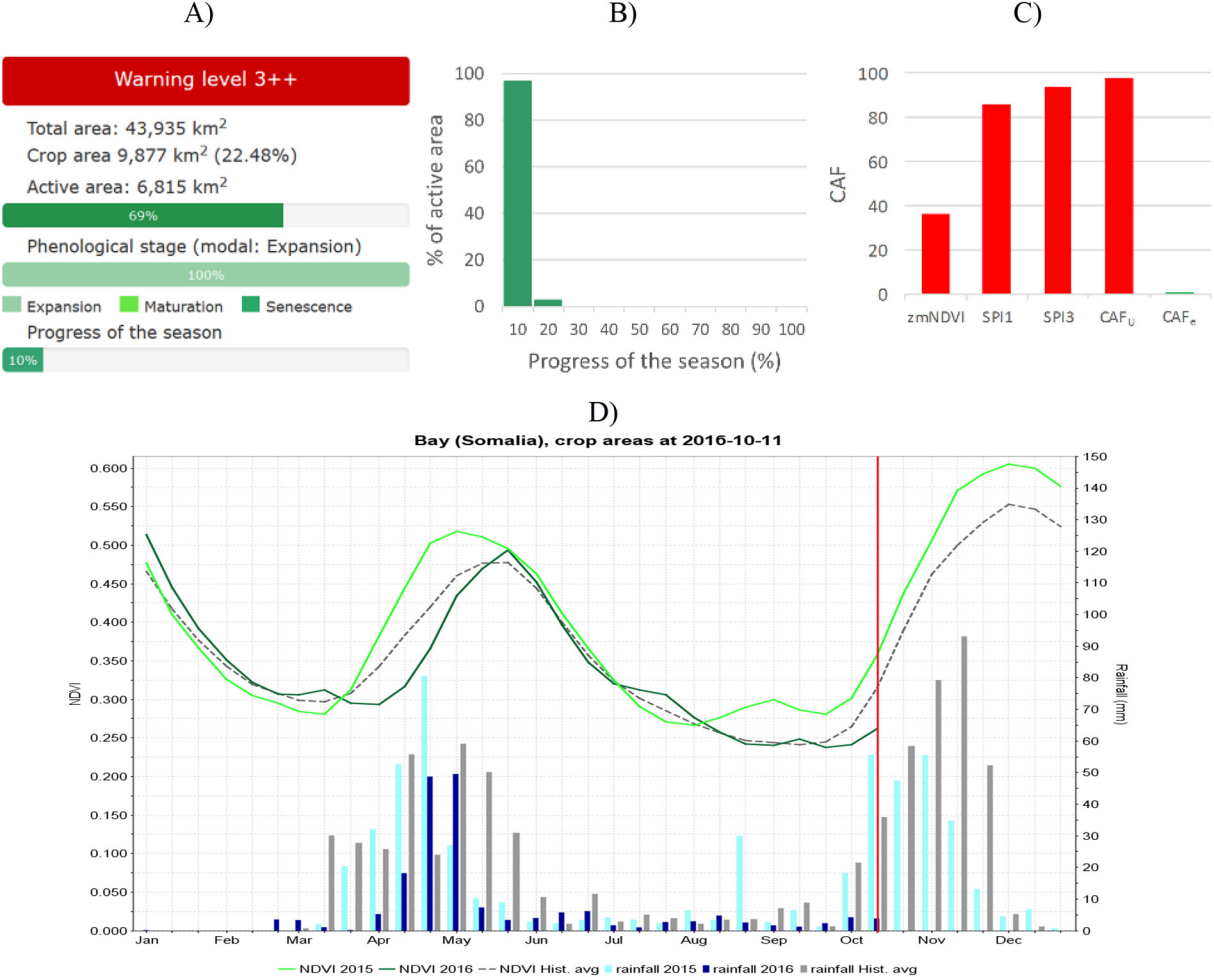
Statistics and graphs generated by the ASAP system on 20-Oct-2016 for the Bay unit of Somalia. A) Warning level details and summary statistics. B) Percentage of active area by progress of the season classes. C) Critical area fraction by indicator. D): Temporal profiles of rainfall and NDVI for the unit. Previous year (2015) and historical average (Hist. avg.) are reported for comparison. The vertical red line indicates the time of analysis (in this case 2nd dekad of October).

For the ASAP unit of Bay, the main production region for rainfed sorghum, Fig. 4A-C reports the detailed information of the warning: the level (3++, thus triggered by the three indicators considered), the area statistics (crop area, active crop, area), the phenology (modal phase, median progress and distribution) and the histogram of the share of active area being affected by the various anomalies. Fig. 4D shows the temporal evolution of rainfall and NDVI for the current year and compares them with the historical average. This additional information extracted from the ASAP system confirm the validity of the warnings (Fig. 3). The profile shows for example that for Bay not only the 2016 *Deyr* season (Oct. to Jan.) is clearly below average, but that the preceding *Gu* season (Apr. to Jul. 2016) had also been affected by a delayed start and poor rainfall early in the season.

**Fig. 5:**
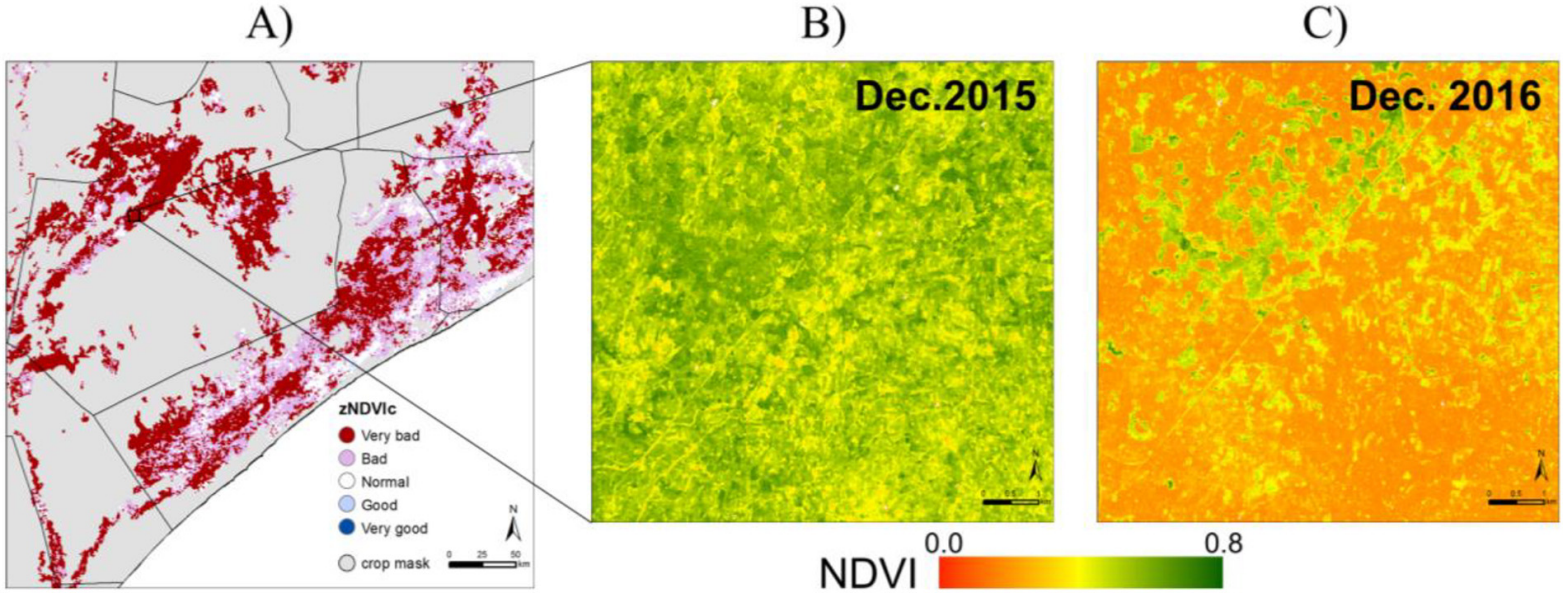
A) ASAP indicator zNDVIc (20 Dec. 2016) for crop areas. The numerical values are: > 1 (Very good), 0.5:1 (Good), −0.5:0.5 (Normal), −1:-0.5 (Bad), and < −1 (Very bad). B) Spatial zoom of a rain-fed agricultural area in unit Bay, Somalia (black box in A) of Sentinel 2 maximum value composite NDVI for the month of December 2015. C) As B) but for December 2016.

Finally, Fig. 5 shows an example of the high resolution analysis provided by the ASAP web tool based on Google Earth Engine. Fig. 5A shows the zNDVIc indicator at 1 km spatial resolution for the cropland area of central Somalia as of the 20 Dec. 2016 while Fig. 5B and C shows the 10 m resolution NDVI monthly maximum composite for a spatial subset (black square polygon in Fig. 5A, Bay unit) obtained from Sentinel 2 instruments for December 2015 and 2106, respectively. NDVI is computed from Level-1C products of Sentinel 2A and B (top of atmosphere reflectances) in Google Earth Engine.

A clear reduction in NDVI is visible for December 2016 as compared to December 2015. Relatively high value NDVI areas in December 2016 (green colours in Fig. 5C) are related to shrublands, which maintain a close to normal vegetation vigour. The rest of the area (mostly in orange) is occupied by rain-fed agriculture and shows reduced NDVI values for the season.

The first ASAP analyst assessment took place in October 2016. Using the automatic warnings and the high resolution evidence as input, the analysts classified Kenya and Somalia as minor and major agricultural production hotspot country, respectively. This difference was due to the fact that Coastal Kenya is a marginal area for the crop production of the entire country, while in Somalia the main producing areas were affected. In the following months, Kenya was also classified as a major hotspot since the drought conditions extended to other areas in the country, including the pastoral drylands in the North.

The onset and evolution of the drought conditions as depicted by ASAP warning was then confirmed by the Food and Nutrition Assessment Unit of Somalia (FSNAU, 2017a) that declared a critical food security situation (i.e. high risk of IPC phase 5 - famine) for the country on the 2nd of February. After that, persistent drought conditions were depicted by ASAP warnings in Southern Somalia and coastal areas of Kenya. The various analyses performed for this season and the seasonal forecasts pointing to below average rainfall expectations affecting also the main season in Somalia (April–June), led to a joint statement of JRC, FEWSNET, WFP and FAO on the 21st of February. This document provided detailed information about the evolution and expected impacts of the drought (WFP et al., 2017). Critical food security and the need of humanitarian assistance were repeatedly confirmed in the following months and persisted at the time of writing this manuscript (July 2017, FSNAU, 2017b).

## 6 Ways forward

A number of improvements have been developed and are currently being tested. The cropland and rangeland masks have been updated using an optimal region-specific selection of recent available global and regional land cover products (Pérez-Hoyos et al., 2017a; Pérez-Hoyos et al., 2017b).

The MetOp NDVI time series has been replaced by the Moderate Resolution Imaging Spectroradiometer (MODIS) NDVI filtered for optimal noise removal in near real-time applications (Klisch and Atzberger, 2016), a processing that reduces the uncertainty and noise contamination in near real-time NDVI data. MODIS has also replaced SPOT-VEGETATION for the calculation of the phenological parameters. Copernicus biophysical products (e.g. the Fraction of Absorbed Photosynthetically Active Radiation, FAPAR; https://land.copernicus.eu/global/themes/vegetation) at 1 km spatial resolution and 10-day temporal frequency form the reprocessed SPOT-VEGETATION archive (Toté et al., 2017) and Proba-V observations are now available and could be used as a back-up in the case of MODIS failure.

Additional improvements have been already scheduled and will become available in 2018. In particular, the SPI1 indicator will be replaced by the standardized anomaly of the Global Water Requirement Satisfaction Index, a soil water balance model conceptually similar to that of Popov and Frere (1986) and aligned with the ASAP phenology. In addition, the ASAP system will integrate information coming from feedback provided by the food security analyst community and will extend the set of indicators provided to analysts and users as ancillary information. Among these indicators, the Heat Magnitude Day index (Zampieri et al., 2017) will be implemented to monitor the effect of heat waves, shown to have a significant impact on agricultural production worldwide. Finally, we plan to replace the current ECMWF precipitation data with the new ECMWF reanalysis product ERA5 (ECMWF, 2017). Compared to the current settings in which we use different models for the historical archive (ERA-Interim) and the NRT (HRES) data, ERA5 will deliver homogeneous data with 31 km spatial resolution.

System updates are announced in the “news” section of the website (https://mars.jrc.ec.europa.eu/asap/news.php) and documented in the updates of the ASAP manual (https://mars.jrc.ec.europa.eu/asap/asap-info.php).

## 7 Conclusions

Since 2001 MARS has developed agricultural monitoring methods for food security early warning outside Europe and has produced in house, or contributed together with other agencies, to a long series of food security assessments and bulletins. More recently, different global remote sensing based products and monthly expert analysis have been combined into a new early warning platform for the identification of agricultural production hotspot countries on a monthly basis. The hotspot country assessment, the ASAP unit level anomaly warnings and the weather anomaly data used, do directly feed into multi-agency global crop monitoring efforts such as the GEOGLAM Crop Monitor activities (for Early Warning and for the Agricultural Market Information System, AMIS), and provide inputs to more detailed food security assessments such as those implementing the IPC/Cadre Harmonisé framework (IPC Global Partners, 2012; AGRHYMET, 2014).

The hotspot assessment is largely based on the described automated warning classification system, that monitors crops and rangelands status at a 10-day time step, globally and in near real-time. But it also includes analysis of other multi-resolution remote sensing indicators and of other information sources such as media monitoring.

During recent major droughts that affected Southern Africa in 2015–2016 and the Horn of Africa in 2016–2017, the ASAP automatic warnings were found to be timely and accurate in detecting onset and spatial extent and the concerned countries have been identified as agricultural production hotspots in a timely manner.

The ASAP system has been tested since mid-2016 and has been officially launched at the European Development Days (EDD) in Brussels in June 2017, and is since then used in a fully operational mode. The selection and processing of input indicators is constantly under improvement, as is the online visualization platform.

## References

[bb0005] AGRHYMET (2014). Cadre Harmonisé manual: Identification and Analysis of Areas at Risk and Populations Affected by Food and Nutrition Insecurity in the Sahel and West Africa.

[bb0010] Atzberger C., Vuolo F., Klisch A., Rembold F., Meroni M., Marcio Pupin M., Formaggio A., Thenkabail P.S. (2016). Agriculture. Remote Sensing Handbook.

[bb0015] Baruth B. (2018). What does it entail to run a European crop monitoring system? A retrospective analysis of the past 25 years. Agric. Syst..

[bb0020] Bicheron P., Defourny P., Brockmann C., Schouten L., Vancutsem C., Huc M., Bontemps S., Leroy M., Achard F., Herold M., Ranera F., Arino O. (2008). GLOBCOVER: product description and validation report. Technical Report.

[bb0025] Bosard M., Feranec J., Otahel J. (2000). CORINE land Cover Technical Guide - Addendum 2000. European Environmental Agency Technical Report No. 40.

[bb0030] Bureau of Rural Sciences of Australia (2006). User Guide and Caveats for the 1992/93, 1993/94, 1996/97, 1998/99, 2000/01 and 2001/02 Land Use of Australia, Version 3. Technical report.

[bb0035] Colombo R., Meroni M., Busetto L., Rossini M., Panigada C., Thenkabail P.S., Lyon J.G., Huete A. (2011). Optical remote sensing of vegetation water content. Hyperspectral Remote Sensing of Vegetation.

[bb0040] Core Team R. (2016). R: A Language and Environment for Statistical Computing.

[bb0045] ECMWF (2015). User Guide to ECMWF Forecast Products. https://www.ecmwf.int/sites/default/files/elibrary/2015/16559-user-guide-ecmwf-forecast-products.pdf.

[bb0050] ECMWF (2017). ERA5 data documentation. https://software.ecmwf.int/wiki/display/CKB/ERA5+data+documentation.

[bb0055] Eerens H., Haesen D., Rembold F., Urbano F., Tote C., Bydekerke L. (2014). Image time series processing for agriculture monitoring. Environ. Model. Softw..

[bb0060] FAO (1993). Land cover of Afghanistan. FAO Project Number AFG/97/001/a/08/12. Available Online.

[bb0065] FAO (2014). The Global Administrative Unit Layers (GAUL) 2014. http://www.fao.org/geonetwork/srv/en/metadata.show?currTab=simple&id=12691.

[bb0070] FAO (2016). 2015–2016 El Niño - Early Action and Response for Agriculture, Food Security and Nutrition - UPDATE #10.

[bb0075] FAO GIEWS (2017). Prolonged and severe drought exacerbates food insecurity. Special Alert No. 339, East Africa.

[bb0080] Food Security Information Network (2017). Global Report on Food Crisis. https://www.wfp.org/content/global-report-food-crisis-2017.

[bb0085] Fritz S., Laso Bayas J.C., See L., Waldner F., Jacques D., Becker-Reshef I., Whitcraft A., Baruth B., Bonifacio R., Crutchfield J., Rembold F., Rojas O., Van der Velde M., Verdin J., Wu B., Yan n., Gilliams S., Mucher S., Moorthy I., McCallum I. (2018). A comparison of global agricultural monitoring systems and current information gaps. Agric. Syst..

[bb0090] FSNAU (Food and Nutrition Assessment Unit) (2017). Nearly 3 million people in Somalia face crisis and emergency acute food insecurity risk of famine increases. http://www.fsnau.org/downloads/FSNAU-FEWSNET-Technical-Release-February-2017.pdf.

[bb0095] FSNAU (Food and Nutrition Assessment Unit) (2017). Quarterly brief - focus on post Gu 2017 season early warning. http://fsnau.org/in-focus/quarterly-brief-june-2017-focus-post-gu-season-early-warning.

[bb0100] Funk C., Budde M.E. (2009). Remote sensing of environment Phenologically-tuned MODIS NDVI-based production anomaly estimates for Zimbabwe. Remote Sens. Environ..

[bb0105] Funk C., Peterson P., Landsfeld M., Pedreros D., Verdin J., Shukla S., Husak G., Rowland J., Harrison L., Hoell A., Michaelsen J. (2015). The climate hazards infrared precipitation with stations—a new environmental record for monitoring extremes. Sci. Data.

[bb0110] Genovese G. (2004). Methodology of the mars crop yield forecasting system. EUR Report EUR 21291 EN/1–4.

[bb0115] GEOGLAM (2016). Early Warning Crop Monitor No. 5 - June 2016. https://cropmonitor.org/index.php/2016/06/03/crop-monitor-for-early-warning-june-2016.

[bb0120] Giri C., Jenkins C. (2005). Land cover mapping of greater Mesoamerica using MODIS data. Can. J. Remote. Sens..

[bb0125] Gorelick Noel, Hancher Matt, Dixon Mike, Ilyushchenko Simon, Thau David, Moore Rebecca (2017). Google earth engine: planetary-scale geospatial analysis for everyone. Remote Sens. Environ..

[bb0130] Holben B.N. (1986). Characteristics of maximum-value composite images from temporal AVHRR data. Int. J. Remote Sens..

[bb0135] Homer C., Huang C., Yang L., Wylie B., Coan M. (2004). Development of a 2001 National Landcover Database for the United States. Photogramm. Eng. Remote. Sens..

[bb0140] IPC Global Partners (2012). Integrated Food Security Phase Classification Technical Manual Version 2.0. Evidence and Standards for Better Food Security Decisions.

[bb0145] Klisch A., Atzberger C. (2016). Operational drought monitoring in Kenya using MODIS NDVI time series. Remote Sens..

[bb0150] Massart M., Rembold F., Rojas O., Leo O., Chuvieco E., Li J., Yang X. (2010). The Use of Remote Sensing Data and Meteorological Information for Food Security Monitoring, Examples in East Africa. Advances in Earth Observation of Global Change.

[bb0160] Meroni M., Marinho E., Sghaier N., Verstrate M., Leo O. (2013). Remote sensing based yield estimation in a stochastic framework — case study of durum wheat in Tunisia. Remote Sens..

[bb0165] Meroni M., Fasbender D., Kayitakire F., Pini G., Rembold F., Urbano F., Verstraete M.M. (2014). Early detection of biomass production deficit hot-spots in semi-arid environment using FAPAR time series and a probabilistic approach. Remote Sens. Environ..

[bb0170] Meroni M., Verstraete M., Rembold F., Urbano F., Kayitakire F. (2014). A phenology-based method to derive biomass production anomalies for food security monitoring in the horn of Africa. Int. J. Remote Sens..

[bb0175] Middleton N., Thomas D. (1997). World Atlas of Desertification.

[bb0180] Min. of Agriculture, Forestry and Fisheries (2016). Summer Crops: Final Production Estimate (2016) and Winter Cereals: Revised Area Planted and Second Production Forecast, South Africa. http://www.nda.agric.za/docs/Cropsestimates/Media%20Sept%202016.pdf.

[bb0185] Nemani R.R., Keeling C.D., Hashimoto H., Jolly W.M., Piper S.C., Tucker C.J., Myneni R.B., Running S.W. (2003). Climate-driven increases in global terrestrial net primary production from 1982 to 1999. Science.

[bb0190] Obe R., Hsu L. (2011). PostGIS in Action.

[bb0195] Pérez-Hoyos A., Rembold F., Gallego J., Schucknecht A., Meroni M., Kerdiles H., Leo O., Kayitakire F. (2017). Development of a new harmonized land cover/land use dataset for agricultural monitoring in Africa. WorldCover 2017 Conference, 14–16 March 2017.

[bb0200] Pérez-Hoyos A., Rembold F., Kerdiles H., Gallego J. (2017). Comparison of global land cover datasets for cropland monitoring. Remote Sens..

[bb0205] Popov G., Frere M. (1986). Early agrometeorological crop yield assessment. FAO Plant Production and Protection Paper 73.

[bb0210] Prince S.D. (1991). Satellite remote sensing of primary production: comparison of results for Sahelian grasslands 1981–1988. Int. J. Remote Sens..

[bb0215] Python Core Team (2016). Python: A Dynamic, Open Source Programming Language.

[bb0220] Rahman H., Dedieu G. (1994). SMAC: a simplified method for the atmospheric correction of satellite measurements in the solar spectrum. Int. J. Remote Sens..

[bb0225] Rembold F., Meroni M., Urbano F., Royer A., Atzberger C., Lemoine G., Eerens H., Haesen D., Aidco D.G., Klisch A. (2015). Remote sensing time series analysis for crop monitoring with the SPIRITS software: new functionalities and use examples. Front. Environ. Sci..

[bb0230] Rembold F., Meroni M., Atzberger C., Ham F., Fillol E., Thenkabail P.S. (2016). Agricultural drought monitoring using space-derived vegetation and biophysical products: a global perspective. Remote Sensing Handbook.

[bb0235] Rembold F., Kerdiles H., Lemoine G., Perez-Hoyos A. (2016). Impact of El Niño on agriculture in southern Africa for the 2015/2016 main season. Technical Report.

[bb0240] Rojas O., Rembold F., Royer A., Negre T. (2005). Real-time agrometeorological crop yield monitoring in eastern Africa. Agron. Sustain. Dev..

[bb0245] Rojas O., Vrieling A., Rembold F. (2011). Assessing drought probability for agricultural areas in Africa with coarse resolution remote sensing imagery. Remote Sens. Environ..

[bb0250] Rouse J.W., Haas R.H., Schell J.A., Deering D.W., Harlan J.C. (1974). Monitoring the Vernal Advancements and Retro Gradation of Natural Vegetation.

[bb0255] Steinberger R., Lupu M., Kanoulas E. (2013). Multilingual and cross-lingual news analysis in the Europe media monitor (EMM). Multidisciplinary Information Retrieval. 6th 1059 Information Retrieval Facility Conference.

[bb0260] Swets D., Reed B.C., Rowland J.D., Marko S.E. (1999). A weighted least-squares approach to temporal NDVI smoothing. Proceedings of the 1999 ASPRS Annual Conference. American Society of Photogrammetric Remote Sensing, Prtland, Oregon.

[bb0265] Toté C., Swinnen E., Sterckx S., Clarijs D., Quang C., Maes R. (2017). Evaluation of the SPOT/VEGETATION collection 3 reprocessed dataset: surface reflectances and NDVI. Remote Sens. Environ..

[bb0270] Tucker C.J., Vanpraet C.L., Sharman M.J., Van Ittersum G. (1985). Satellite remote sensing of total herbaceous biomass production in the Senegalese Sahel: 1980–1984. Remote Sens. Environ..

[bb0275] Vancutsem C., Marinho E., Kayitakire F., See L., Fritz S. (2013). Harmonizing and combining existing land cover/land use datasets for cropland area monitoring at the African continental scale. Remote Sens..

[bb0280] Volante J.N. (2009). Monitoreo de la cobertura y el uso del suelo a partir de sensores remotos. INTA technical report project PNECO.

[bb0285] Warmerdam F., Hall B., Leahy M.G. (2008). The geospatial data abstraction library. Open Source Approaches in Spatial Data Handling.

[bb0290] WFP VAM (2017). East Africa: The 2016 Season: Severe Drought in the Horn of Africa. http://documents.wfp.org/stellent/groups/public/documents/ena/wfp289530.pdf.

[bb0295] WFP, FEWS NET, European Commission, FAO (2017). Persistent drought in Somalia leads to major food security crisis. Joint Multi Agency Statement.

[bb0300] WFP VAM (2016). Southern Africa: Growing Season 2015–2016: A Season of Regional Drought. http://documents.wfp.org/stellent/groups/public/documents/ena/wfp282670.pdf.

[bb0305] White M.A., Thornton P.E., Running S.W. (1997). A continental phenology model for monitoring vegetation responses to interannual climatic variability. Glob. Biogeochem. Cycles.

[bb0310] World Meteorologic Organization (2012). Standardized Precipitation Index User Guide.

[bb0315] Zampieri M., Ceglar A., Dentener F., Toreti A. (2017). Wheat yield loss attributable to heat waves, drought and water excess at the global, national and subnational scales. Environ. Res..

